# Preserflo MicroShunt Outcomes in Rare and Refractory Glaucoma Entities Compared with Matched Primary Open-Angle Glaucoma Controls: A Retrospective Exploratory Study

**DOI:** 10.2147/OPTH.S599778

**Published:** 2026-08-13

**Authors:** Emil Nasyrov, Martin Kowalski, Lasse Wolfram, Caroline J Wenzel, Bogomil Voykov

**Affiliations:** 1 Centre for Ophthalmology, University Hospital Tuebingen, Tuebingen, Germany

**Keywords:** neovascular glaucoma, post-traumatic glaucoma, angle-closure, primary congenital glaucoma, aphakia, juvenile open-angle glaucoma

## Abstract

**Purpose:**

To investigate the efficacy and safety of the Preserflo MicroShunt (PMS) in rare and refractory glaucoma entities (RRG) in comparison with primary open-angle glaucoma (POAG).

**Patients and Methods:**

In this retrospective exploratory study, the primary outcome was the complete 1-year success rate (≥20% reduction in IOP from baseline and ≤18 mmHg with no medication use). Kaplan–Meier estimates were compared between RRG and POAG (matched 1:1) using the log-rank test. Exploratory sub-analyses were performed for each RRG subgroup. The secondary outcomes included bleb revision and complication rates.

**Results:**

Eighty eyes were included in the RRG group (8 primary congenital (PCG), 11 juvenile open-angle (JOAG), 10 aphakic (AG), 14 combined mechanisms (CMG), 17 neovascular (NVG), 13 post-traumatic (TG), 4 oil-induced, 2 steroid-induced glaucoma, and 1 with idiopathically elevated episcleral venous pressure) and matched to 80 eyes with POAG based on baseline characteristics. The 1-year success rates were 56% in the RRG and 71% in the POAG groups (p = 0.0079). Exploratory subgroup analyses revealed significantly lower success in PCG (2/8), TG (8/13) and NVG (8/17). Success rates in the other groups were not significantly different from POAG. Bleb revisions were required significantly more frequently in the RRG group (58% vs 41%). Severe complications were observed in the RRG group, but did not reach significance.

**Conclusions:**

While the PMS demonstrated lower efficacy in RRG compared to POAG overall, this difference was primarily attributable to worse outcomes in NVG, TG, and PCG. Success rates in AG, JOAG, and CMG were not significantly different from POAG controls. The frequent need for postoperative bleb revisions, particularly in NVG, PCG, and TG subgroups, represents an important practical consideration. Given the small subgroup sizes and exploratory design, these findings warrant validation in larger, prospective studies.

## Introduction

Minimally invasive bleb surgery (MIBS) procedures using the Preserflo MicroShunt (PMS) have demonstrated a comparable efficacy to trabeculectomy and a favourable safety profile in primary open-angle glaucoma (POAG).^1^ The therapeutic potential has also been investigated in common secondary glaucoma entities such as pseudoexfoliative glaucoma (PXG).^2^ However, reports for less common glaucoma entities are few. The outcomes of the PMS in glaucoma secondary to anterior uveitis has been demonstrated to depend on the uveitis aetiology.^3^ Glaucoma secondary to vitrectomy and silicon-oil has been effectively treated with PMS in the short term of 6 months.^4^ Overall, the efficacy and safety of the PMS for rare glaucoma entities, that are refractory to previous glaucoma surgery or IOP-lowering medications, remain under-investigated, and its therapeutic potential in these cases remains to be determined.

Rare and refractory glaucoma (RRG) entities encompass a heterogeneous group of conditions with markedly different underlying pathophysiological mechanisms, which may differentially influence surgical outcomes following bleb-forming procedures.^5^,^6^ Factors such as conjunctival fibrosis from prior surgical interventions, chronic inflammatory activity, altered aqueous humor dynamics, and neovascularization can impair bleb survival and compromise long-term IOP control.^5^,^7^ For instance, neovascular glaucoma is characterised by aggressive fibrovascular proliferation driven by vascular endothelial growth factor (VEGF) that may accelerate bleb failure, whereas post-traumatic glaucoma may involve angle recession and conjunctival scarring that alter filtration dynamics.^8–11^ In congenital glaucoma, unique developmental anatomical features of the anterior segment, including buphthalmos, altered limbal anatomy, and an exuberant fibrotic postoperative response, may predispose to filtration failure.^12^,^13^ These mechanistic differences highlight the importance of subgroup-specific outcome data.

The PMS may offer potential advantages over trabeculectomy or glaucoma drainage devices (GDD) in selected refractory glaucoma subtypes. Its ab externo subconjunctival approach avoids intraocular manipulation, reducing the risk of hypotony-related complications and the surgical complexity associated with tube shunt procedures.^14^,^15^ Long-term data have demonstrated sustained IOP reduction up to 5 years following PMS implantation with no long-term sight-threatening adverse events.^16^,^17^ Postoperative bleb management strategies, including needling and incisional revision, may further modulate outcomes and represent an important consideration when evaluating the practical benefits of MIBS in complex cases.^18^,^19^

This study aimed to evaluate the efficacy and safety of the PMS in RRG entities, that have not been previously reported, in comparison with a matched POAG control to identify cohorts that could benefit from PMS implantation.

## Methods

This retrospective exploratory study considered primary glaucoma entities other than POAG and normal-tension glaucoma and secondary glaucoma entities other than PXG and pigment dispersion glaucoma to be eligible for inclusion in the RRG group. Uveitic glaucoma types were not included in this study, as we have previously published outcomes of the PMS in different uveitis subtypes.^3^ Thus, eyes with primary congenital glaucoma (PCG), juvenile open-angle glaucoma (JOAG), combined mechanism glaucoma (CMG), aphakic glaucoma (AG), neovascular glaucoma (NVG), post-traumatic glaucoma (TG), oil-induced glaucoma (OiG), steroid-induced glaucoma, and glaucoma secondary to idiopathically elevated episcleral venous pressure (EVP) were included in the RRG group. Notably, the RRG cohort is inherently heterogeneous, encompassing conditions with markedly different mechanisms, inflammatory status, fibrosis risk, and postoperative management requirements. This heterogeneity limits the interpretation of pooled analyses, and subgroup results should be considered exploratory.

CMG was defined as POAG with coexisting angle-closure features that do not fulfil the criteria for angle-closure glaucoma and may present with features such as prior intermittent angle closure or angle closure attack which had resolved after successful peripheral laser iridotomy or cataract surgery with persistently elevated IOP.^20^

Eyes with RRG were matched to POAG controls in a 1:1 ratio regarding clinical baseline characteristics. Eyes with and without previous glaucoma or cataract surgeries were eligible for inclusion. The exclusion criteria were a follow-up period of less than 6 months. The study was conducted in accordance with the tenets of the Declaration of Helsinki. The requirement for patient consent for data to be used in this study was waived by the ethics committee of the University Hospital Tuebingen due to its retrospective design (project number 074/2023BO2). All patient data were handled in accordance with applicable data protection legislation and institutional policies. Patient confidentiality was strictly maintained, and all data were anonymised prior to analysis and reporting.

### Surgical Technique and Preoperative Management

Implantation of the PMS was performed by a senior glaucoma surgeon (BV) under topical anaesthesia, as described previously.^3^ At the time the indication for surgery was made, preoperative IOP was assessed under maximal tolerated IOP-lowering medication. Topical medication was then stopped 2 weeks before surgery. Oral acetazolamide 250 mg was started up to three times daily and stopped on the day before surgery. Unpreserved dexamethasone eye drops were started three times per day one week before surgery, and were applied five times per day starting from postoperative day 1 and were then tapered over 6–8 weeks. Starting on the first postoperative day, moxifloxacin eye drops were applied four times per day for 2 weeks. Follow-ups were conducted on postoperative days 1 and 14; at postoperative months 3, 6, 9, and 12 and then twice a year. These involved a full ophthalmological examination, which included IOP measurement using Goldmann applanation tonometry, slit-lamp examination, and fundus examination.

### Postoperative Bleb Management

Conjunctival bleb needling was performed in case of IOP elevation above the target range, typically >21 mmHg, associated with bleb encapsulation or Tenon cyst formation in the absence of other identifiable causes of IOP rise. Incisional bleb revision was indicated either when needling failed to restore adequate filtration or was performed primarily in cases of a dense subconjunctival fibrosis, suggested by the PMS not being visible under the conjunctiva. The decision to perform needling vs incisional revision was made by the treating surgeon (BV) based on clinical assessment of bleb morphology, IOP trajectory, and response to prior interventions. The indications and thresholds for intervention remained consistent throughout the study period.

### Study Outcome Measures and Baseline Characteristics

The primary outcome measure was the complete surgical success after 1 year. Success was defined as a ≥20% reduction in IOP from baseline with an IOP of ≤18 mmHg (Category 2), in accordance with the guidelines of the World Glaucoma Association (WGA).^21^ Instead of a lower IOP threshold for the definition of success (eg >6 mmHg), hypotony was defined clinically by the presence of choroidal effusion or hypotony maculopathy, and its absence was defined as a success criterion.^22^ If patients did not meet the IOP and hypotony success criteria at two consecutive visits starting from month 3, failure was recorded at the first visit in which the criteria were not met. Furthermore, loss of light perception, the need for further glaucoma surgery, including incisional bleb revisions, and the need for oral acetazolamide were considered failures. Needling procedures were not regarded as failures according to the WGA and European Glaucoma Society Guidelines.^21^,^23^ Additional success categories were considered for a ≥25% reduction in IOP from baseline with an IOP of ≤15 mmHg (Category 1) and a ≥20% reduction in IOP from baseline with an IOP of ≤21 mmHg (Category 3). Complete success was considered without topical IOP-lowering medication use. Qualified success A was defined as the achievement of the success criteria regardless of whether topical IOP-lowering medication was used (the same or a smaller number of agents compared with before surgery). For qualified success B one incisional revision was allowed additionally. The secondary outcome measures included the median IOP compared with baseline, frequency of medication-free eyes, intervention rates (including needlings, incisional bleb revisions, or further glaucoma surgeries), and complication rates.

Patients’ characteristics, histories, and clinical outcome measurements were retrieved from an electronic medical record system. Autorefraction was performed using the ARK-1s autorefractor (NIDEK, Gamagori, Japan) to assess refractive error. The spherical equivalent refractive error of phakic patients was retrieved from visits where no cataract was noted. Conversely, the refractive error of pseudophakic patients was obtained from visits prior to cataract surgery.

### Matching

Eyes in the RRG group were matched to those with POAG in a 1:1 ratio using propensity score matching. Propensity scores were estimated via a logistic regression model, where RRG served as the dependent variable and a set of clinically relevant covariates as the independent variables. The covariates used for matching were age, sex, preoperative IOP, number of preoperative medications, best corrected visual acuity (BCVA), spherical equivalent of refractive error, pseudophakia, previous glaucoma surgical procedures, and potential follow-up period. Nearest neighbour matching without replacement was applied to select one POAG control for each RRG eye. Post-matching balance between the groups was assessed using standardised mean differences (SMD) to ensure comparability across the measured covariates. An SMD of 0.2 was considered indicative of adequate balance. Supplemental Figure 1 depicts the distribution of SMDs before and after matching. Despite propensity score matching, residual imbalances persisted in age, BCVA, and aphakia status between the RRG and POAG groups. These imbalances reflect the inherent clinical characteristics of the RRG cohort (eg, younger age in PCG and JOAG, aphakia in AG) that could not be fully balanced without excluding clinically relevant cases. Alternative matching strategies, including caliper-based matching, were considered but were not pursued due the risk of excessive case exclusion. Additionally, several factors that might influence filtering surgery outcomes, including conjunctival scarring severity, ischemic status, inflammatory activity, prior anti-VEGF treatment, angle configuration, and prior vitrectomy, could not be reliably quantified or standardised from retrospective clinical records and were therefore not included in the matching model. The entire matching procedure was implemented using the MatchIt package in R (R Foundation for Statistical Computing, Vienna, Austria).

### Statistical Analysis

Descriptive statistics were used to summarise the demographic and clinical characteristics of patients. For continuous variables, normality was assessed using the Shapiro–Wilk test, which did not indicate a normal distribution of continuous patient characteristics or outcomes. These variables were reported as medians and interquartile ranges (IQRs). Categorical variables, such as the requirement of revisional surgeries, were summarised as percentages. The number of revisions per patient was calculated per patient-year of follow-up. In-group comparisons for continuous variables were performed using the Wilcoxon matched-pairs signed-rank test for non-normally distributed data. Comparisons between groups for continuous variables were conducted using the Mann–Whitney *U*-test for non-normally distributed data. For categorical variables, the chi-square test or Fisher’s exact test was used, as appropriate. Kaplan–Meier survival analyses of success categories were used and compared using the log-rank test, with group-wise survival probabilities reported at 6, 12, 18 and 24 months. Subgroup analyses were performed for each RRG entity with a minimum number of 4 eyes. Both eyes from 9/71 patients in the RRG and 8/72 patients in the POAG group were included in the analyses. This may violate the assumption of independence underlying the statistical tests and survival analyses used. To assess the potential impact of inter-eye correlation, a sensitivity analysis was performed including only the first eye per patient (Supplemental Figure 3). Given the exploratory nature of subgroup analyses and limited sample sizes across several glaucoma entities, no adjustment for multiple comparisons was applied. The statistical findings from subgroup analyses should be interpreted cautiously and considered primarily hypothesis-generating. All analyses were conducted using R version 4.4.0 (R Foundation for Statistical Computing) with the survival and survminer packages and GraphPad Prism version 10.2.0 (GraphPad Software, Boston, USA). A probability value of p < 0.05 was considered statistically significant.

## Results

### Study Eyes

Table 1 shows the demographic and clinical characteristics of the RRG and POAG groups. In the RRG group all eligible eyes were included resulting in 80 eyes of 71 patients. These included 8 eyes (6 patients) with PCG, 11 eyes (8 patients) with JOAG, 10 eyes (9 patients) with AG, 14 eyes (12 patients) with CMG, 17 eyes (16 patients) with NVG, 13 eyes (13 patients) with TG, 4 eyes (4 patients) with OiG, 2 eyes (2 patients) with steroid-induced glaucoma, and 1 eye (1 patient) with glaucoma secondary to idiopathically elevated EVP. The aetiology of NVG was proliferative diabetic retinopathy in 11 cases, venous occlusion in 6 cases and occlusive vasculitis of unknown origin in 1 case. The RRG eyes were matched to 80 eyes (72 patients) with POAG out of 343 possible matches. The baseline parameters were similar between the matched groups, except for a significantly lower age and worse best-corrected visual acuity (BCVA) logMAR in the RRG group compared with the POAG control. Significantly more eyes with aphakia were included in the RRG group, due to the nature of this study, while the number of phakic eyes were similar between groups (Table 1). Characteristics of the subgroups are summarised in Supplemental Table 1. The AG, JOAG and PCG subgroups had a significantly lower age compared with the POAG control.

**Table 1:** Demographic and Clinical Characteristics and Surgical History of Patients Receiving the PMS

Characteristic	RRG n = 80 Eyes of 71 Patients	POAG n = 80 Eyes of 72 Patients	*p*-value
Median age (IQR), year	56 (37–64)	60 (51–67)	**0.0014**^**a**^
Female sex, %	30	33	0.8647^b^
White ethnicity, %	90	92.5	0.07807^a^
African	3.7	1.3	
Middle Eastern	3.7	2.5	
South-Asian	1.3	0	
East-Asian	1.3	3.7	
Lens Status, %			**0.0069**^**c**^
Phakic	27.5	26	>0.9999^b^
Pseudophakic	61	74	
Aphakic	11	0	
Laterality, % right eye	50	49	> 0.999^b^
Median refractive error (IQR), dpt spherical equivalent	0 (−2.5 to 0)	−0.75 (−3 to 0)	0.2061^a^
Median BCVA (IQR), logMAR	0.4 (0.1−0.7)	0.1 (0−0.4)	**0.0018**^**a**^
Median Mean Deviation (IQR), dB	−10.4 (−18.4 to −4.5)	−8.4 (−17.6 to −3.5)	0.4585^a^
Median global ppRNFLT (IQR), µm	68 (49−81)	59 (50−75)	0.1717^a^
Median preoperative medicated IOP (IQR), mmHg	29 (24–36)	28 (24–34)	0.2754^a^
Median number of preoperative medications (IQR)	3 (3–4)	3 (3–4)	0.9013^a^
Median follow-up time (IQR), months	18 (6–29)	20 (8–32)	0.5073^a^
**Previous glaucoma procedures, %**			0.8977^c^
None	43	50	
Laser trabeculoplasty	10	13	
Cyclo-coagulation	15	9	
Angle-based procedures	10	9	
XEN-45 implantation	7	6	
Trabeculectomy	11	10	
Ahmed Glaucoma Valve	3	3	

**Notes**: Preoperative IOP and medications at the time of indication for surgery. The number of medications used was calculated based on the individual active agents. Statistical tests: ^a^Mann–Whitney *U*-test; ^b^Fisher’s exact test; ^c^chi-square test. P-values < 0.05 are bolded.

**Abbreviations**: RRG, rare and refractive glaucoma entities; BCVA, best-corrected visual acuity; IQR, interquartile range; PMS, Preserflo MicroShunt; POAG, primary open-angle glaucoma; ppRNFLT, peripapillary retinal nerve fibre layer thickness; SD, standard deviation.

### Postoperative IOP Changes After PMS Implantation

**Figure 1:**
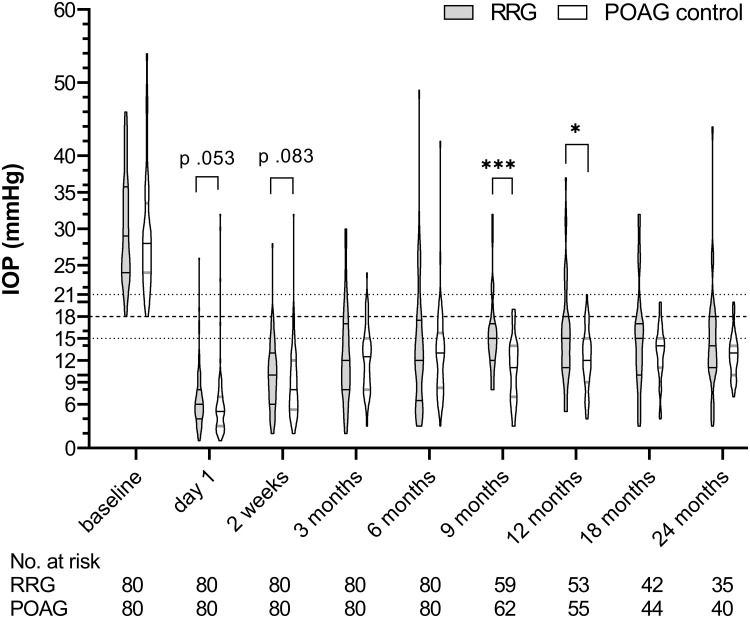
Postoperative IOP evolution after PMS implantation in RRG and matched POAG eyes. Postoperative median (IQR) IOP is plotted across postoperative visits (violin plot indicating the 95% confidence intervals). The RRG group is represented in grey and the POAG group in white. Horizontal dotted lines indicate relevant cut-off values regarding the success criteria. Groups were compared using the Mann–Whitney U-test: * = p < 0.05; *** = p < 0.001.

The median IOP and number of medications were significantly reduced from baseline at all visits until 2 years after PMS implantation in both the RRG and POAG groups (each p < 0.001; Wilcoxon matched-pairs signed-rank test; Figure 1). The median (IQR) IOP at months 6, 12 and 24 were 12 (7–18), 15 (11–18), and 14 (11–18) mmHg in the RRG group and 13 (8–16), 12 (9–15), and 13 (10–14) mmHg in the POAG group, respectively (Figure 1). Median IOP was significantly higher at month 9 and 12 in the RRG group, with trends indicating a higher IOP in the early postoperative period at day 1 and 14 (Mann–Whitney test; Figure 1). The median (IQR) number of medications at months 6, 12 and 24 was reduced to 0 (0–0) in both RRG and POAG groups (each p < 0.0001 compared to baseline; Wilcoxon matched-pairs signed-rank test). In the RRG group, 90%, 88%, and 86% of the eyes were medication-free at months 6, 12, and 24, respectively. The corresponding values in the POAG group were 99%, 98%, and 95%, respectively (p = 0.0339, p = 0.052 and p = 0.2349 at each visit, respectively, compared with the RRG group; Fisher’s exact test). Supplemental Figure 2 depicts the postoperative IOP at 6 and 12 months for each eye compared to baseline values.

### Surgical Success of the PMS for RRG vs POAG

**Figure 2:**
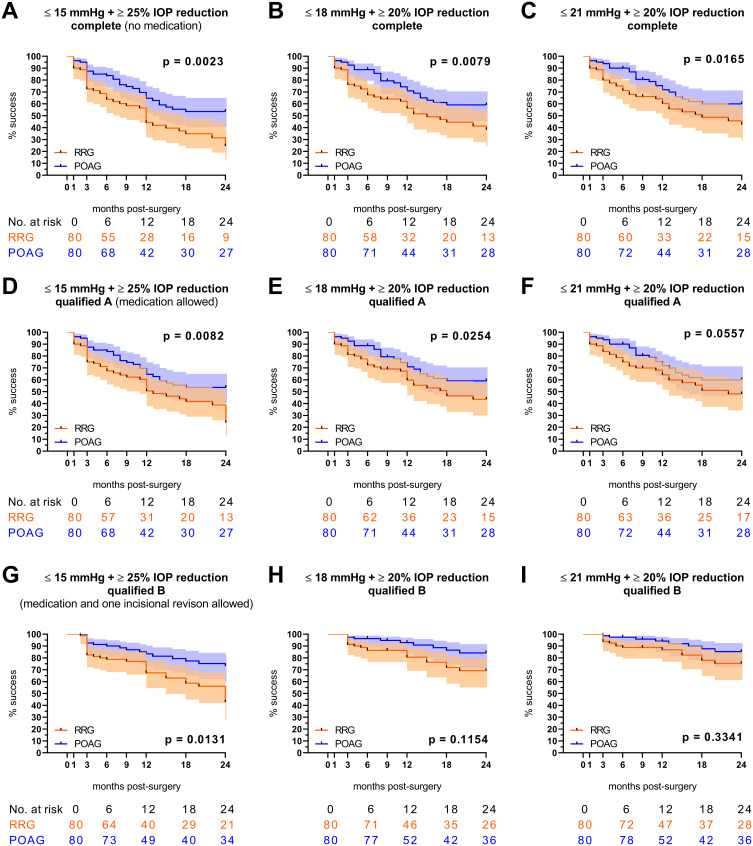
Kaplan–Meier survival estimates of complete and qualified success after PMS implantation in RRG and matched POAG eyes. Kaplan–Meier estimates were used to compare complete success, not allowing incisional revisions or medication use to achieve target IOP (**A**–**C**), qualified success A, allowing for medication use not exceeding baseline (**D**–**F**) and qualified surgical success B allowing for one incisional revision (**G**–**I**) between eyes with RRG (orange) and POAG (blue) using the log-rank test. Eyes that did not meet IOP success criteria or had clinical hypotony at two consecutive visits were considered a failure or were censored at last follow-up (vertical ticks). The 95% confidence intervals are represented in lighter orange and blue colours, respectively.

Figure 2 depicts the Kaplan–Meier survival estimates of the complete and qualified success rates. Supplemental Table 2 summarises the success rates (95% CI) at different postoperative visits. A sustained IOP of ≤15 mmHg with a ≥25% IOP reduction without medication (complete success for category 1) was significantly lower in the RRG group, achieved by 44% (95% CI = 32–56) at year 1 compared with 65% (52–75) in the POAG group (p = 0.0023; log-rank test; Figure 2A). Success rates for qualified success A and B were also significantly lower (p = 0.0082 and 0.0131, respectively, Figure 2D and G, Supplemental Table 2).

Complete success for category 2 (IOP of ≤18 mmHg with a ≥20% IOP reduction) was significantly lower in the RRG group compared with the POAG group, achieved by 56% (44–67) and 71% (58–80), respectively (p = 0.0079; log-rank test; Figure 2B). Success rates were significantly lower for qualified success A, allowing for medication use (p = 0.0254), but not qualified success B, allowing additionally for one incisional revision (p = 0.1154, Figure 2E and H, Supplemental Table 2).

Complete success for category 3 (IOP of ≤21 mmHg with a ≥20% IOP reduction) was significantly lower in the RRG group than the POAG group, achieved by 60% (48–71) and 72% (59–81), respectively (p = 0.0204; log-rank test; Figure 2C, Supplemental Table 2). Qualified success rates A and B were similar between groups (p = 0.0557 and p = 0.3341, respectively, Figure 2F and I, Supplemental Table 2).

Sensitivity analyses, including only the first operated eye per patient, were performed revealing similar success rates and significant differences between groups persisted for all complete success categories (Supplemental Figure 3). A difference for qualified success A was indicated by a trend for categories 2 as compared with the all-eyes analysis.

### Exploratory Analyses of Surgical Success of the PMS in RRG Subgroups

RRG subgroups, with at least 4 eyes included, were analysed and exploratorily compared with the complete POAG control. Table 2 summarises the success rates (95% CI) at different postoperative visits for success category 2 (IOP ≤ 18 mmHg and ≥ 20% reduction). Success rates for the other IOP targets (categories 1 and 3) are presented in Supplemental Table 3. These subgroup analyses are exploratory and should be interpreted with caution given the small sample sizes (n < 10 in PCG OiG, steroid-induced glaucoma and glaucoma secondary to idiopathic elevation of EVP).

**Table 2:** Exploratory Analyses of Success Following PMS Implantation in RRG Subgroups Compared to the POAG Control

	PCG	JOAG	AG	CMG	TG	NVG	OiG
	**IOP ≤ 18 mmHg and ≥ 20% reduction** **Complete success % (no medication, no incisional revision)**						
month							
6	38 (9–67)	73 (37–90)	80 (41–95)	86 (54–96)	62 (31–82)	65 (38–82)	75 (21–62)
12	25 (4–56)	73 (37–90)	58 (23–82)	86 (54–96)	62 (31–82)	45 (21–67)	75 (21–62)
18	25 (4–56)	73 (37–90)	58 (23–82)	57 (9–88)	37 (10–65)	30 (10–54)	75 (21–62)
24	25 (4–56)	0	58 (23–82)	57 (9–88)	25 (4–54)	20 (4–45)	75 (21–62)
***p***	**0.0031**	0.6965	0.8792	0.9980	**0.0282**	**0.0028**	0.7665
	**Qualified success A % (medication allowed)**						
month							
6	38 (9–67)	91 (51–99)	80 (41–95)	86 (54–96)	62 (31–82)	76 (49–90)	75 (21–62)
12	25 (4–56)	91 (51–99)	58 (23–82)	86 (54–96)	62 (31–82)	51 (25–72)	75 (21–62)
18	25 (4–56)	76 (30–94)	58 (23–82)	57 (9–88)	37 (10–65)	36 (14–59)	75 (21–62)
24	25 (4–56)	38 (1–80)	58 (23–82)	57 (9–88)	25 (4–54)	36 (14–59)	75 (21–62)
***p***	**0.0038**	0.9198	0.9792	0.9980	**0.0282**	**0.0390**	0.8216
	**Qualified success B % (medication and one incisional revision allowed)**						
month							
6	50 (15–77)	100	100	93 (59–99)	92 (57–99)	82 (55–94)	75 (21–62)
12	50 (15–77)	100	100	93 (59–99)	92 (57–99)	63 (36–82)	75 (21–62)
18	50 (15–77)	100	100	93 (59–99)	66 (26–88)	55 (28–76)	75 (21–62)
24	50 (15–77)	67 (5–95)	100	93 (59–99)	66 (26–88)	55 (28–76)	75 (21–62)
***p***	**0.0448**	0.5352	0.1819	0.9705	0.6029	*0.0578*	0.6141

**Notes**: Kaplan-Meier success rates with (95% Confidence intervals) are presented. P-values were calculated using the Log-rank test for each depicted subgroup compared with the POAG control group. P-values <0.05 are bolded and <0.1 are highlighted in italics. Success rates of the POAG control group are summarised in Table 2.

**Abbreviations**: PCG, primary congenital glaucoma; JOAG, juvenile open-angle glaucoma; AG, aphakic glaucoma; CMG, combined mechanism glaucoma; TG, post-traumatic glaucoma; NVG, neovascular glaucoma; OiG, oil-induced glaucoma; PMS, Preserflo MicroShunt.

Among all subgroups, the PCG (n = 8) group had the lowest success rates regarding category 2 (IOP of ≤18 mmHg with a ≥20% IOP reduction). Complete and qualified success rates were significantly lower compared with the POAG group. The TG (n = 13) and NVG (n = 17) groups had the second lowest success rates among the subgroups, which were significantly lower (complete and qualified A) compared to POAG eyes. Success rates in the other investigated subgroups such as eyes with AG (n = 10), CMG (n = 14), OiG (n = 4) and JOAG (n = 11) were not significantly different from the POAG control.

The 2 eyes with steroid-induced glaucoma from a 72-year-old female and 76-year-old male patient, respectively, achieved complete success across categories 1–3 at the end of follow-up after 6 months. IOP was decreased from 26 and 42 mmHg at baseline to 10 and 13 mmHg, respectively. The 2 eyes with steroid-induced glaucoma were medication free after 6 months.

Regarding the rates of medication free eyes in the other subgroups, these were 100% in the AG and CMG groups at 6, 12 and 24 months, respectively. Corresponding rates were 85%, 88% and 75% in the JOAG group; 80%, 88% and 83% in the PCG group; 90%, 78% and 83% in the NVG group; 93%, 100% and 88% in the TG group; and 75%, 100%; and 100% in the OiG group, respectively.

### PMS Implantation in Glaucoma Secondary to Idiopathic Elevation of EVP

This is the first report of a PMS implantation in a 54-year-old male patient with glaucoma secondary to idiopathic elevation of EVP (Radius-Maumenee syndrome). Other causes of elevated EVP were ruled out by extensive diagnostic work-up. IOP was 32 mmHg under 4 topical medications and oral acetazolamide 250 mg 3 times per day at baseline and decreased to 18 mmHg and 16 mmHg after 6 and 12 months after PMS implantation without medication use, respectively. Postoperative choroidal effusion developed despite normotonic IOP and required a viscoelastic injection into the AC at day 3 and intraluminal placement of a 10–0 nylon suture at day 8, resolving the effusion by day 18. The suture was removed at day 170 due to increasing IOP, however, resulting in choroidal effusion. A 10–0 nylon suture was placed intraluminally at day 175, resolving the effusion. Incisional revision was performed at day 284 and 344 due to bleb fibrosis and a suture was prophylactically placed inside the PMS lumen. BCVA logMAR was 0 at baseline and 0.1 at the 1-year visit.

### Complications and Revisions After PMS Implantation

Table 3 summarises the complication and revision rates between the RRG and POAG groups following PMS implantation. The rates of clinical hypotony, constituting choroidal effusion, and anterior chamber (AC) haemorrhage and were similar between the groups. The intervention rates for choroidal effusions were comparable, and all effusions resolved after a median of 13 days in both groups.

**Table 3:** Complication and Bleb Revision Rates Following PMS Implantation in RRG and Matched POAG Eyes

Complication, % (Eyes)	RRG n = 80 Eyes	POAG n = 80 Eyes	*p*-value
Choroidal effusion at any visit	21.3 (17)	23.8 (19)	0.8501^a^
AC formation with viscoelastic	11.3 (9)	12.5 (10)	>0.9999^a^
Intraluminal stenting	1.3 (1)	2.5 (2)	>0.9999^a^
Median days to resolution of choroidal effusion	13	13	0.8917^b^
AC haemorrhage	40 (32)	46.3 (37)	0.5233^a^
Endophthalmitis	1.3 (1)	0	>0.9999^a^
Malignant glaucoma (aqueous misdirection)	2.5 (2)	0	0.4969^a^
Dislocation of intraocular lens	3.8 (3)	0	0.2453^a^
Corneal decompensation	2.5 (2)	1.3 (1)	>0.9999^a^
Loss of BCVA **≥** 2 lines at last follow up	23.8 (19)	21.3 (17)	0.8501^a^
Further glaucoma surgery	10 (8)	2.5 (2)	0.0983^a^
**Bleb revision rates within complete follow-up, % (eyes)**			
Any bleb revision	58 (47)	41.3 (33)	**0.0407**^**a**^
Needling	46.3 (37)	25 (20)	**0.0080**^**a**^
Incisional revision	40 (32)	35 (28)	0.6224^a^
Median Needlings (IQR) per patient	0 (0−1)	0 (0−0.75)	**0.0035**^**c**^
Median incisional revisions (IQR) per patient	0 (0−1)	0 (0−1)	0.6092^c^
**Bleb revision rates within 6 months, %**			
Any bleb revision	31.3 (25)	15 (12)	**0.0236**^**a**^
Needling	22.5 (18)	10 (8)	0.0523^a^
Incisional revision	18.8 (15)	8.8 (7)	0.1065^a^
**Bleb revision rates within 12 months, %**			
Any bleb revision	42.5 (34)	30 (24)	0.1385^a^
Needling	35 (28)	20 (16)	0.0508^a^
Incisional revision	27.5 (22)	22.5 (18)	0.5842^a^
**Number of bleb revisions per patient year follow-up**			
Any bleb revision	0.809	0.494	
Needling	0.453	0.205	
Incisional revision	0.356	0.289	

**Notes**: Statistical tests: ^a^Fisher’s exact test; ^b^Log-rank test, ^c^Mann–Whitney-*U*-test. P-values < 0.05 are bolded.

**Abbreviations**: AC, anterior chamber; PMS, Preserflo MicroShunt; POAG, primary open-angle glaucoma; RRG, rare and refractive glaucoma entities.

Severe complications such as endophthalmitis were observed in one eye with PCG after 345 days. *Staphylococcus epidermidis* was isolated by diagnostic vitrectomy and successfully treated with intravitreal vancomycin. Loss of BCVA from 1.1 to 2.0 logMAR was noted. Malignant glaucoma (aqueous misdirection) was observed in 1 eye with CMG and 1 eye with NVG requiring iridectomy with anterior vitrectomy after 2 and 24 days, respectively, which successfully relieved aqueous misdirection in both cases. Corneal decompensation was observed in 1 PCG eye and 1 TG eye with previous Ahmed glaucoma valve (AGV) implantation, requiring DMEK and perforating keratoplasty after 1 and 2 years, respectively. In the control group 1 eye with previous AGV implantation also required listing for DMEK. Dislocation of an intraocular lens was observed in 1 eye with AG (sclera-fixated) and 2 eyes with TG.

Overall, loss of 2 lines BCVA logMAR or more was found in 17 POAG eyes (21%) and 19 RRG eyes (24%; p = 0.8501, Fisher’s exact test). Rates of eyes with loss of 2 lines or more were highest among PCG (5/8), steroid-induced glaucoma (1/2) and NVG (8/17). Rates were similar or lower compared to the POAG control in OiG (1/4), AG (1/10), JOAG (2/11), CMG (1/14) and TG (0/13). However, no loss-of light perception was observed in any eye.

The rates of any bleb revision (needling or incisional) were significantly higher in the RRG group within the first 6 months and within the complete follow-up (Table 3). Needlings were required significantly more in the RRG group in the complete follow-up, with trends at 6 and 12 months. Regarding the RRG subgroups, overall bleb revision rates within the complete follow-up were highest in NVG (13/17), followed by PCG (6/8), TG (8/13) and AG (6/10) and comparable to the POAG control in OiG (2/4), steroid-induced glaucoma (1/2), JOAG (5/11) and CMG subgroups (4/14). Needlings were required more frequently in NVG (11/17), followed by AG (6/10), PCG (4/8), OiG (2/4), steroid-induced glaucoma (1/2), JOAG (4/11), TG (4/13) and CMG (4/14). Incisional revisions were required in decreasing order more frequently in PCG (5/8), TG (7/13), steroid-induced glaucoma (1/2), NVG (7/17), AG (4/10), JOAG (3/11), OiG (1/4), and CMG (3/14).

There was a trend towards more secondary glaucoma surgery in the RRG group (p = 0.0983). Two eyes in the POAG group compared to 4 eyes in the RRG group (1 eye each with CMG, JOAG, TG, steroid-induced glaucoma, respectively) received another PMS implantation within the follow-up. Additionally, one eye with PCG received cyclo-cryoablation. Two eyes with NVG and 1 eye with JOAG required cyclo-photoablation.

## Discussion

Our study found that the success of the PMS was significantly lower in eyes with RRG compared with eyes with POAG. The RRG group included refractory cases of primary glaucoma (PCG, JOAG, CMG) and secondary glaucoma entities (AG, TG, NVG, OiG, steroid-induced glaucoma, and Radius-Maumenee syndrome). This is the first study presenting outcomes of the PMS in subgroups of PCG, JOAG, and AG as well as NVG, TG, CMG and the first case report of the PMS for Radius-Maumenee syndrome.

Overall, 56% of the RRG group compared to 71% in the POAG group achieved a sustained IOP <18 mmHg with a ≥ 20% reduction from baseline, without medication use or requirement for incisional bleb revisions, after 1 year. In the exploratory subgroup-analyses significantly worse outcomes were found in eyes with PCG, NVG and TG, achieving success rates of 25%, 45% and 62% after 1 year. Outcomes in the JOAG, AG, OiG and CMG subgroups were not significantly different from the POAG control. However, the limited sample sizes, especially in the PCG and OiG subgroups, preclude definitive conclusions. Qualified success, allowing for medication use to achieve the IOP target, was significantly lower in the RRG group, as well as in the PCG, NVG and TG subgroups in the exploratory analyses. However, when allowing for one incisional bleb revision, qualified success rates were not significantly different from the POAG control in the RRG group as well as all subgroups except for eyes with PCG and NVG, the latter indicated by a trend. Considering an IOP target of <21 mmHg with a ≥ 20% reduction, all subgroups, except for PCG eyes, achieved success not significantly different from the POAG control when allowing for medication use and one incisional revision (Supplemental Table 2).

### Postoperative Revision Burden and Complications

In the current study, bleb revision surgery, specifically needlings, were required more frequently in the RRG group compared with the POAG group within the complete follow-up (58% vs 41% and 46% vs 25%). Incisional revisions were comparable between groups (40% vs 35%). In the subgroup-analyses, bleb revision rates were highest in the NVG (13/17, 76%), PCG (6/8, 75%), TG (8/13, 62%) and AG (6/10, 60%) groups, with needlings required most frequently in NVG (11/17), AG (6/10), PCG (4/8) and incisional revisions most frequently in PCG (5/8), TG (7/13) and NVG (7/17). Bleb revision rates were however comparable to the POAG control in JOAG (5/11) and CMG (4/14). Notably, the higher bleb revision rates reflect the worse outcomes in the PCG, TG and NVG subgroups in contrast to lower revision rates and better outcomes in the CMG and JOAG subgroups. Durr et al reported needling rates of 11.8% following PMS implantation in refractory glaucoma eyes, that had already failed at least one subconjunctival filtering surgery, which are lower than the revision rates observed in our RRG cohort, likely reflecting the different composition of refractory cases.^24^

The considerable postoperative revision burden observed in several RRG subgroups warrants careful consideration when evaluating the practical advantages associated with MIBS. Although qualified success rates improved substantially when one incisional revision procedure was permitted, the frequent need for repeated needling and incisional revisions, particularly in NVG, PCG, and TG, implies more frequent postoperative visits and intensive management that may offset some of the practical benefits of a minimally invasive primary procedure. This also has implications for healthcare resource utilisation, patient convenience, and the overall cost-effectiveness of the approach. Notably, the mitomycin C (MMC) dosage of 0.2 mg/mL used in this study might have contributed to overall higher revision burden.^25^ A higher dosage which is associated with lower revision rates in POAG or a longer application (>3 min) may thus be considered in patients with NVG, PCG and TG.^26^ However, long-term effects of higher concentrations of MMC on post-PMS bleb morphology or complications such as late-leakage are not known. Surgeons should weigh the benefit of lower rates of incisional revisions, which are generally found to be safe, against potential long-term risks on an individual basis.^18^,^27^

While complications such as AC haemorrhage or choroidal effusion were self-limiting and comparable between the RRG and POAG groups, more serious complications were observed in the RRG group, however, not reaching significance. Endophthalmitis was successfully treated in one eye with PCG. Aqueous misdirection was observed in 1 eye with CMG and 1 eye with NVG, but successfully resolved in both cases following anterior vitrectomy.

### Glaucoma Entities That Might Have Limited or No Benefit from PMS Implantation

The substantially lower success rates observed in PCG, NVG and TG were associated with the highest rates of bleb revisions and likely reflect the aggressive fibrovascular healing responses and conjunctival scarring characteristic of these conditions.^5^,^9^,^10^,^12^ The primary cause of filtration surgery failure is fibroblast proliferation and subconjunctival fibrosis at the surgical site. In NVG, retinal ischemia results in the release of VEGF, which diffuses into the aqueous and anterior segment, triggering neovascularization and promoting fibrous tissue proliferation, directly accelerating fibrosis and filtration failure.^9^ Furthermore, VEGF upregulation stimulates Tenon’s fibroblast proliferation, and inhibition of VEGF with bevacizumab has been demonstrated to reduce angiogenesis, collagen deposition, and scar formation after filtration surgery in experimental models.^28^ These mechanisms likely explain the high bleb revision rates (76%) and lower success observed in our NVG subgroup. In TG, post-traumatic angle recession has been identified as an independent risk factor for bleb failure after trabeculectomy and prior conjunctival and scleral scarring from the initial trauma and any subsequent surgical interventions may additionally compromise bleb formation and long-term patency.^10^,^29^ Similarly, the poor outcomes in PCG may be attributable to the unique developmental anatomy of the pediatric and young adult eye, including a thicker Tenon capsule, more robust wound healing response, and altered anterior segment architecture, all of which may predispose to filtration failure.^12^,^13^ Buphthalmic eyes present additional technical difficulties due to limbal distortion and altered anterior segment architecture, which may further predispose to filtration failure. These pathophysiological considerations highlight the importance of careful patient selection and the need for intensive postoperative bleb management in these subgroups.

Studies investigating the PMS in rare glaucoma types are few. Kaliche et al found a 100% success rate in 12 patients with OiG after 6 months (IOP ≤ 21 mmHg and ≥ 30% reduction).^4^ This is similar to our findings of a 75% after 6 months in a small cohort of 4 OiG eyes, however using a slightly different success definition (IOP ≤ 21 mmHg and ≥ 20% reduction). We found that success was sustained by month 12. Bleb revision rates of 50% were similar to our findings of 50% (2/4) overall bleb revisions (50% needlings and 25% incisional revisions). The PMS has also been demonstrated as a feasible option for refractory IOP elevation secondary to a intravitreal dexamethasone implant which is in line with our findings in two patients with steroid-induced glaucoma.^30^ Overall our findings for OiG and steroid-induced glaucoma are limited by the small sample size and further prospective, multicentre studies are warranted for this rare entity.

In general, reports for outcomes of filtering procedures in rare glaucoma entities are few and sub-analyses of mixed cohort are rarely reported, due to the low number of eyes included for each glaucoma type. Manners et al performed trabeculectomy with MMC in TG eyes of South-African patients (n = 41) and found a complete success rate (IOP ≤ 21 mmHg) of 85% and 81% at 1 and 2 years, respectively, which is higher compared to 58% and 35% in our TG group (n = 13) after PMS implantation (IOP ≤ 21 mmHg and ≥ 20% reduction, no clinical hypotony).^29^ However, a less strict success definition without considering IOP reduction or hypotony was employed by Manners et al to investigate trabeculectomy which might overestimate true success. Late hypotony complications occurred in no patient with TG after PMS implantation in our study, but in 9% following trabeculectomy in the study by Manners, with 2% constituting chronic hypotony maculopathy.^29^ Also, African decent is a known risk factor in filtering surgery, while most eyes in our TG group were from white patients, making a comparison of results more difficult.^31^

Overall, trabeculectomy with MMC might be more effective in lowering IOP than the PMS in TG. However, the PMS might be a safer option, since our TG group had no severe, hypotony-related, complications and allowing for medication use and one incisional bleb revision (qualified success B) our TG group achieved success rates of 100% and 71% after 1 and 2 years, which are comparable to those found after trabeculectomy.^29^ However, TG eyes had among the highest bleb revision rates (8/13) in our study, with more than half of the patients (7/13) requiring at least one incisional revision. While the PMS might present a feasible alternative, the high revision burden should be considered as a trade-off for possibly lower hypotony-related complications.

For the treatment of NVG, it remains disputed whether trabeculectomy or GDD surgery present the best option regarding filtering surgery.^32–34^ Tokumo et al found 1-year success rates of 59.1 and 61.6% using Baerveldt glaucoma implantation (BGI) vs trabeculectomy in a randomised controlled trial in Japanese patients with NVG (n = 23 and 27, respectively).^33^ Using the same success definition (IOP ≤ 21 mmHg and ≥ 20% reduction) we found a 1-year complete success of 63% following PMS implantation in our NVG cohort (n = 17) which might be comparable to the reported rates of trabeculectomy and BGI, although cross-study comparisons should be interpreted with caution given differences in patient populations and study designs. However, the necessity for revision in the NVG eyes was the highest among all investigated subgroups, with 13 out of 17 requiring bleb revisional surgery in contrast to lower rates found for BGI (1/23) and trabeculectomy (6/27).^33^ Overall, while the PMS was less effective in NVG eyes compared to POAG eyes in our analysis, our results suggest a potentially comparable IOP-lowering effect to GDD and trabeculectomy, with lower rates of late complications but drastically higher bleb revision rates.^33^ Long-term data from the primary tube vs shunt study have demonstrated that both tube shunt surgery and trabeculectomy carry substantial complication and reoperation burdens, which should be considered when comparing the PMS to these established procedures.^35^

Regarding the analysis of PCG eyes, significantly lower success of the PMS across all definitions compared with POAG controls was only found in this subgroup in the current study. However, this exploratory comparison was limited by the low number of 8 eyes as well as both eyes being included from 2 of the 6 patients and should be considered hypothesis-generating and requiring confirmation. Further, comparison to other studies is difficult since our cohort included only eyes with previously failed surgery, including secondary surgery such as trabeculectomy. Still, our results for the PMS in PCG were lower compared to other reports, with only 50% achieving IOP ≤ 21 mmHg and ≥ 20% reduction after 1 and 2 years, despite allowing for medication use and one incisional bleb revision.^36^ The unique anatomical and developmental characteristics of eyes with PCG, including buphthalmos, corneal enlargement, altered limbal anatomy, and a more vigorous wound healing response, may contribute to the higher failure rates observed in this subgroup.^12^,^13^ Overall, the mechanisms of PCG, and patients’ histories and characteristics are highly individual and all factors must be considered for each individual case in order to choose the appropriate surgical approach. Even though matching was performed in our study, significant differences in baseline characteristics such as age remained reflecting the inherent clinical characteristics of the RRG cohort. Younger age is often described as a risk factor for failure of traditional filtering surgery but not yet found a risk factor MIBS outcomes^25^,^37^,^38^ Age might still influence the different outcomes between the PCG cohort and POAG control. However, the JOAG and AG subgroups also had significant age differences compared with the POAG control, but similar success rates. Overall, while age might affect outcomes, it is difficult to distinguish the respective influence of age and glaucoma diagnosis due to younger age being associated with specific glaucoma types.

Another MIBS, the XEN-45 GeL Stent, has been investigated by Lv et al using an ab externo approach in a mixed cohort of 42 eyes with refractory secondary glaucoma, mostly including unspecified uveitic glaucoma, 9 cases with steroid-induced glaucoma, 4 cases with NVG, 4 cases with TG and 3 cases with AG.^39^ We previously reported that outcomes of the XEN-45 were significantly associated with the aetiology of uveitic glaucoma.^40^ Comparison of success rates with the current study is further complicated by different success definitions and numbers of glaucoma subtypes included in both studies with a higher amount of NVG and TG cases, which had worse outcomes, in the current study. However, overall bleb revision rates were comparable between the studies despite a shorter follow-up (mean 13.74 months) compared to the current study.^39^ Our 12-months revision rates might be more comparable to the overall revision rates in the study by Lv et al, which was lower for the PMS. A feasible comparison of our results is, however, not possible.

### Glaucoma Entities That Might Benefit from PMS Implantation

While revision rates were high in the AG group, which were younger than the POAG control, all eyes remained medication free up to 24 months, and success rates were not significantly different from the POAG control. A recent review indicated that ab interno trabeculotomy might be the most appropriate first-line treatment for AG.^41^ In our AG group (n = 10), half of the eyes had previously failed surgery, including ab interno 360° trabeculotomy, and half had no previous surgery. Our results suggest that the PMS may represent a viable option for AG, irrespective of previous glaucoma surgery, although confirmation in larger studies is needed. Notably, the AG subgroup was most affected by the residual post-matching imbalances in age and pseudophakia/aphakia status, yet achieved outcomes comparable to POAG controls, suggesting that these baseline differences did not substantially confound the primary comparison. Furthermore, the proportion of phakic eyes was similar between the RRG and POAG groups, indicating that the imbalance in lens status was driven by the inclusion of aphakic eyes inherent to the AG subgroup rather than a systematic difference in lens status between cohorts.

Interestingly, the JOAG subgroup (n = 11) had similar age differences as the AG group compared with the POAG control, but non-significantly different success and revision rates (3/11) in the exploratory analysis. This suggests that differences in age might not be the driving factor for higher bleb fibrosis in AG and that age-related confounding is unlikely to fully explain the worse outcomes observed in other subgroups such as PCG and NVG. In this line, age has not been found as a risk factor for either PMS or XEN-45 implantation success.^25^,^38^ The outcomes for the JOAG subgroup suggest that it may respond similarly to PMS as POAG eyes. Overall, further prospective, multicentre studies are warranted to evaluate the PMS in these glaucoma entities.

The CMG subgroup achieved the highest success rates among the subgroups and demonstrated low bleb revision rates. Ngyuen et al previously reported that combined cataract surgery with a Hydrus Microstent implantation in CMG/primary angle-closure glaucoma resulted in comparable success rates to POAG patients.^42^ Our findings suggest that MIBS, using the PMS, in mainly pseudophakic (12/14) eyes with CMG may be a viable therapeutic option. Despite suboptimal angle anatomy in these eyes, we observed no occlusion of the PMS lumen or angle closure and aqueous misdirection in only one eye. Thus, the risk of aqueous misdirection following PMS might be lower in CMG than for primary angle-closure glaucoma.^43^ Further studies are needed to assess the role of the PMS in treating CMG.

To our knowledge, this study includes the first report of a PMS implantation in glaucoma secondary to idiopathic elevation of EVP (Radius-Maumenee syndrome).^44^ While IOP could be successfully managed following PMS implantation, clinical hypotony had to be addressed repeatedly in this patient by limiting outflow with an intraluminal nylon suture. This is in line with other reports of filtering surgery such as trabeculectomy or glaucoma drainage devices, which reported choroidal effusion despite normotonic IOP in patients with Radius-Maumenee syndrome.^45–47^

## Limitations

Our study had several limitations. First, the retrospective design is inherently susceptible to selection and information bias. Second, the RRG cohort is highly heterogeneous, encompassing glaucoma entities with markedly different pathophysiological mechanisms, inflammatory profiles, fibrosis risk, and surgical histories. While subgroup analyses were performed, some subgroups were severely underpowered, and the findings should be considered exploratory and hypothesis-generating rather than definitive. In particular, observations regarding PCG (n = 8), OiG (n = 4), steroid-induced glaucoma (n = 2) and elevated EVP (n = 1) cannot support clinically meaningful inference.

Third, despite propensity score matching, clinically relevant baseline imbalances persisted in age, BCVA, and aphakia status, all of which could bias toward worse outcomes in the RRG group. However, the AG and JOAG subgroups, which were most affected by these imbalances, achieved outcomes comparable to POAG controls, suggesting that the observed differences in NVG, TG, and PCG may be primarily driven by disease-specific mechanisms, although residual confounding from measured and unmeasured variables cannot be entirely excluded. Additionally, the proportion of phakic eyes was similar between groups, indicating that the lens status imbalance was driven by the inclusion of aphakic eyes inherent to the AG entity rather than a systematic difference between cohorts. Additionally, variables such as conjunctival scarring severity, inflammatory activity, and prior anti-VEGF treatment could not be reliably quantified from retrospective records and were therefore not included in the matching model. Residual confounding from these unmeasured factors cannot be excluded.

Fourth, both eyes from some patients were included in the analyses, which may violate the assumption of statistical independence underlying the tests and survival analyses used. However, a sensitivity analysis excluding second eyes yielded similar results.

Fifth, numerous subgroup comparisons were performed without adjustment for multiplicity, increasing the risk of type I error. Both significant and non-significant findings in small subgroups may reflect random variation rather than true differences.

Finally, the follow-up period, while sufficient to observe meaningful short- to medium-term outcomes, may not capture late failures or complications. Longer-term prospective studies with larger, more homogeneous cohorts are needed to validate these preliminary findings.

## Conclusions

While the PMS demonstrated lower overall efficacy in RRG compared to POAG, exploratory subgroup analyses suggest that this difference was primarily attributable to worse outcomes in NVG, TG, and PCG. Success rates in AG, JOAG, and CMG were not significantly different from POAG controls, although the small sample sizes limit the strength of these comparisons. The frequent need for postoperative bleb revisions, particularly in NVG (76%), PCG (75%), and TG (62%), represents an important practical consideration that may offset some advantages of minimally invasive bleb surgery in these subgroups. Severe complications, while infrequent, were observed exclusively in the RRG group. These exploratory findings suggest that the PMS may have a role in selected RRG entities but require validation in larger, prospective studies before definitive conclusions regarding efficacy and safety can be drawn.
